# Assessment of concordance between fresh-frozen and formalin-fixed paraffin embedded tumor DNA methylation using a targeted sequencing approach

**DOI:** 10.18632/oncotarget.18296

**Published:** 2017-05-30

**Authors:** Bruce Moran, Sudipto Das, Dominiek Smeets, Gillian Peutman, Rut Klinger, Bozena Fender, Kate Connor, Matthias Ebert, Timo Gaiser, Jochen HM Prehn, Orna Bacon, Elaine Kay, Bryan Hennessy, Verena Murphy, Bauke Ylstra, Diether Lambrechts, Annette T. Byrne, William M. Gallagher, Darran P. O’Connor

**Affiliations:** ^1^ Department of Molecular and Cellular Therapeutics, Royal College of Surgeons in Ireland, Dublin, Ireland; ^2^ Department of Oncology, Laboratory of Translational Genetics, VIB Center for Cancer Biology, Leuven, Belgium; ^3^ Department of Oncology, Laboratory of Translational Genetics, Department of Oncology, KU Leuven, Leuven, Belgium; ^4^ OncoMark Ltd., NovaUCD, Belfield Innovation Park, Dublin, Ireland; ^5^ Department of Physiology and Medical Physics, Royal College of Surgeons in Ireland, Dublin, Ireland; ^6^ Department of Internal Medicine, University of Heidelberg, Mannheim, Germany; ^7^ Department of Pathology, Beaumont Hospital, Dublin, Ireland; ^8^ Cancer Trials Ireland, Dublin, Ireland; ^9^ Department of Pathology, VU University Medical Center, Amsterdam, The Netherlands; ^10^ Cancer Biology and Therapeutics Laboratory, UCD School of Biomolecular and Biomedical Science, UCD Conway Institute, University College, Dublin, Ireland

**Keywords:** targeted bisulfite sequencing, methylation, epigenetics, FFPE, tumor preservation

## Abstract

DNA methylation is altered in many types of disease, including metastatic colorectal cancer. However, the methylome has not yet been fully described in archival formalin-fixed paraffin embedded (FFPE) samples in the context of matched fresh-frozen (FF) tumor material at base-pair resolution using a targeted approach. Using next-generation sequencing, we investigated three pairs of matched FFPE and FF samples to determine the extent of their similarity. We identified a ‘bowing’ pattern specific to FFPE samples categorized by a lower CG proportion at the start of sequence reads. We have found no evidence that this affected methylation calling, nor concordance of results. We also found no significant increase in deamination, measured by C>T transitions, previously considered a result of crosslinking DNA by formalin fixation and a barrier to the use of FFPE in methylation studies. The methods used in this study have shown sensitivity of between 60-70% based on positions also methylated in colorectal cancer cell lines. We demonstrate that FFPE material is a useful source of tumor material for methylation studies using targeted sequencing.

## INTRODUCTION

Epigenetic modification including DNA methylation is regarded as one of the factors that regulate gene expression across a variety of diseases including cancer [1–4]. Genome-wide DNA methylation studies involving extensive patient cohorts have demonstrated that malignant neoplastic diseases, such as colorectal cancer, display a significant degree of heterogeneity in their epigenome [5–8]. However, the majority of studies which used FFPE as their primary sample source also used array-based technologies to assess global DNA methylation levels, as opposed to next-generation sequencing (NGS) and despite the technological advancement within this area [9–17].

Advantages of archival FFPE-derived cohorts include availability of extensive clinical, histological information and potentially longitudinal sampling, not necessarily available otherwise. However, this sample type has not been extensively used to generate high-resolution single base DNA methylation profiles with NGS, and this may have resulted in some trepidation in considering this option. We believe this to be partly due to the fact that FFPE-derived DNA presents several challenges in terms of overall quality as well as artifacts associated with preservation. Double-stranded DNA (dsDNA) quality and overall yield have been reported as limiting factors associated with FFPE based samples [18–22]. Similarly, research of the inherent effects of formalin fixation on dsDNA has illustrated that denaturation occurs at AT-rich regions, which results in further chemical interactions such as hydrolysis of the phosphodiester bonds, causing fragmentation [18, 23–25].

In addition, technical issues associated with the protocols commonly used in methylation studies such as bisulfite conversion are also evident. Efficient bisulfite conversion involves exposure of dsDNA to low pH levels and high temperatures, increased duration of which have been shown to increase fragmentation [26]. It has been suggested that fragmentation can result in significantly lower amounts of dsDNA for sequencing experiments [27]. Another issue, regarded as one of the primary artifacts associated with FFPE-derived DNA, involves increased levels of C>T or G>A transitions, introduced as a result of the addition of adenine instead of guanosine due to deamination [18, 28], although previous research suggests targeted sequencing approaches may not suffer from this as much as amplicon based approaches [29]. The fact remains that because C>T events are the premise for assessing occurrence of DNA methylation, this issue might cause difficulty in terms of data interpretation [30].

**Figure 1:**
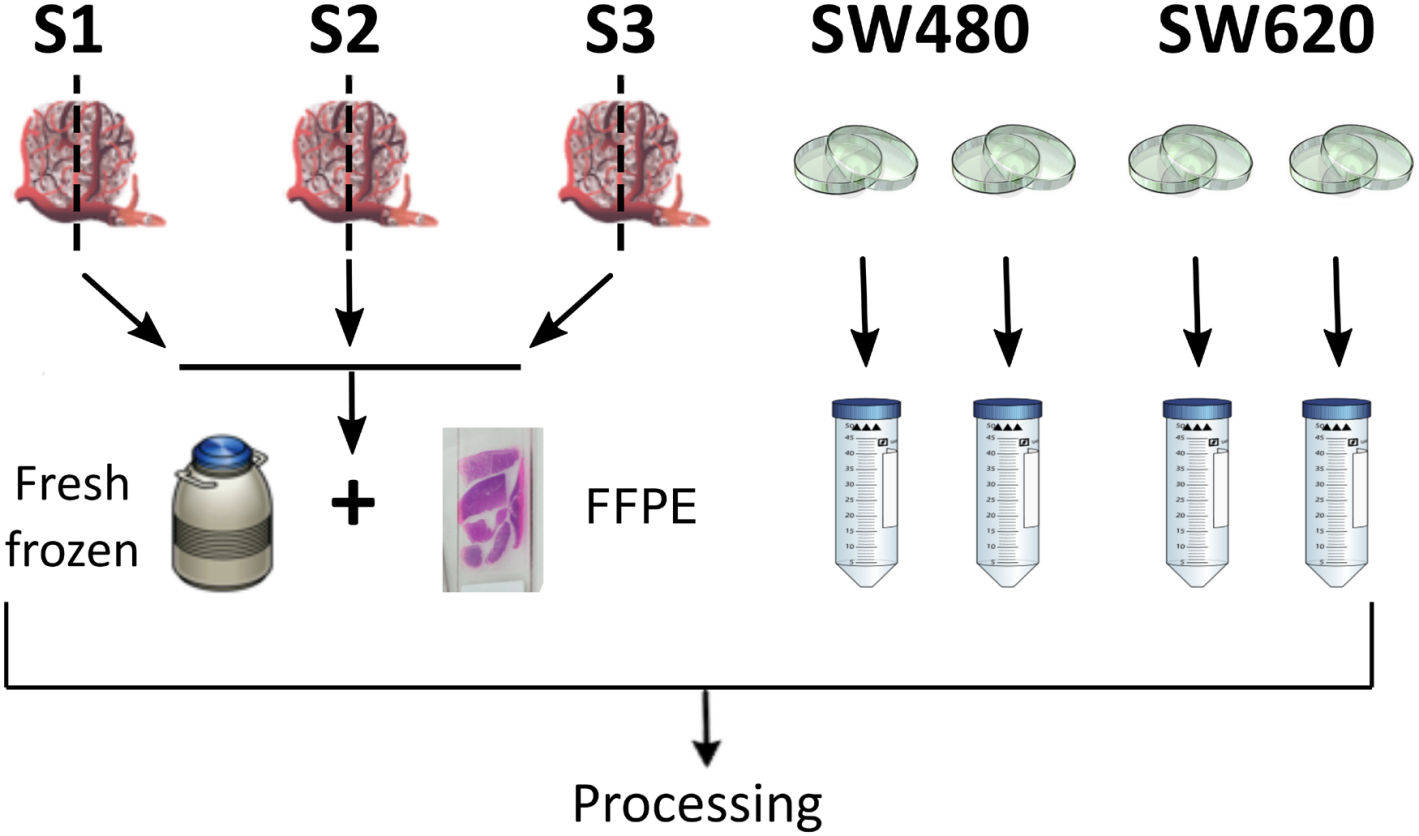
Schematic representation of experimental design and sampling procedure Tumor biopsies S1, S2, S3 were divided and stored using fresh-frozen or FFPE protocols; SW480 and SW620 cells lines were grown in biological duplicate using standard procedures; all biopsy and cell line DNA was then processed identically.

We have applied a targeted sequencing method (SeqCap Epi, Roche) [31, 32] to patient-matched FFPE and FF colorectal cancer tissues, as well as to two colorectal cancer cell lines (Figure 1). Preliminary quality control resulted in the discovery of an FFPE-specific event that we term ‘bowing’. To determine the potential effect of bowing we investigated the level of sequence found ‘off-target’ (i.e. outside the target regions captured) and if it might affect FFPE data in relation to FF. We have found increased levels of C>T transition mutations, indicative of deamination, in one of the samples. However, the increase was found in FF material, and therefore we do not find support for increased deamination in FFPE. We used methylation categories (hypo-, hyper-methylation) using cell line data to identify true and false positives. From this data, we assessed sensitivity of the two preservation types by coverage. Finally, using a mass spectrometry-based approach (Sequenom EPITYPER), we successfully validated several loci that demonstrated both concordance and discordance between FFPE- and FF-derived DNA as determined from our sequencing data. The current study represents an assessment of the utility of bisulfite conversion and the SeqCap Epi system and demonstrates that these methods can be applied to FFPE archival material for methylation analysis.

## RESULTS AND DISCUSSION

### The ‘bowing’ effect

**Figure 2:**
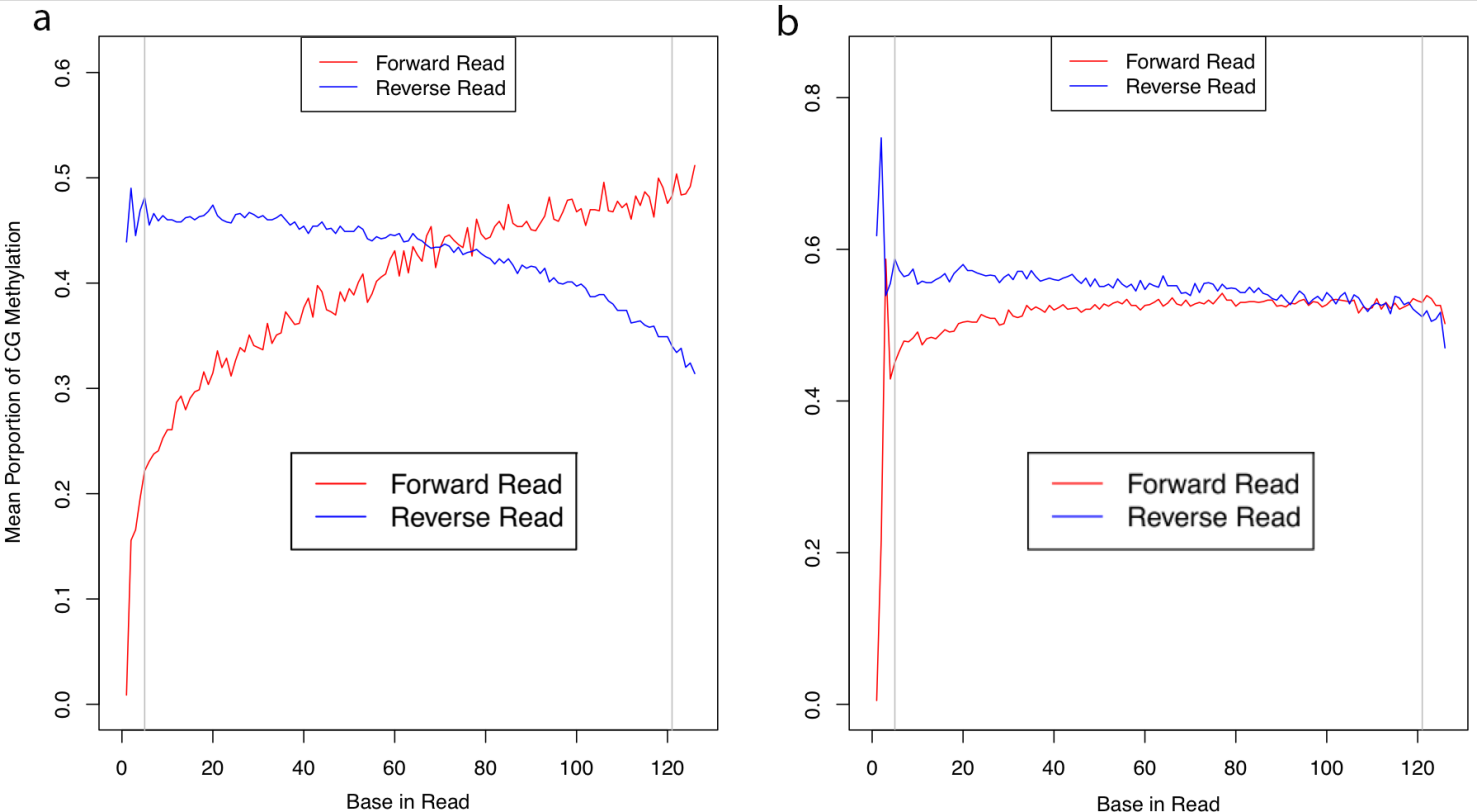
Methylation bias plots Sequence reads from FFPE material **(A)** showed reduced mean CpG methylation proportion at the 5’ end of reads (red line); fresh-frozen material **(B)** did not suffer from this “bowing” effect to the same extent as the FFPE counterpart.

A primary indication of bisulfite conversion and sequence quality used in our analysis were ‘bias-plots’ which show CG methylation proportion per base across sequence reads (Figure 2, Supplementary Figure 1). Our FFPE samples had a very distinctive ‘bowing’ pattern indicating a lower mean proportion of CG methylation at the start of sequence reads within the first 30-40 bases which then resolved to ‘normal’ level (based on FF samples) to within the range of 40-60%. Initially, we believed bowing to be due to adapter contamination. A known issue with FFPE material is increased DNA fragmentation which can result in template DNA which is shorter than the number of bases being sequenced [18]. To eliminate these issues, we conducted computational removal of (i) adapter sequence as it will be sequenced and (ii) as a consequence of adapter read-through on the 3’ end, a duplicated portion of paired-end reads [32]. However, bowing was not resolved following these processes, and given that Illumina adapters are methylated, bisulfite-conversion should result in a proportional methylation level similar to that found in the template DNA.

In this study, we were interested in the use of FFPE for targeted methylation sequencing. We therefore tested if bowing had an impact on the concordance of methylation events between preservation type within samples. We called methylation events (see Methods) and trimmed between 5 and 90bp from the 5’ end in 5bp increments (i.e. not using data from any read before this position in the read for methylation calling). The 5bp level is recommended by the authors of the bwa-meth aligner used [33]. This is due to large deviations in the mean proportion of methylation, which is apparent in all samples (see Figure 2 panel B). Methylation metrics for trimming levels of 5, 30 and 60bp are given in Supplementary Table 1. We tested the concordance of methylation events called at each of the levels of trimming to determine if this corrected any effect that bowing might have had in reducing concordance, and therefore if increased trimming could resolve any impact of bowing.

We used total methylation events in FFPE and the concordant intersection with FF material. If the event was called in both preservation types, and also had the same methylation threshold status in both (i.e. was hypo-, hyper- or intermediately methylated, see *Methylation Calling* in Methods) it was counted as being concordant. We expected that if bowing had an effect of either reducing the apparent incidence of methylation, or of altering the appearance of methylation, increased trimming would result in better concordance after removing the first 30-40 bases where the effect is evident. In that case, correlation would be reduced in these data. Our results show very highly significant correlation of concordance (Spearman's Ro = 1, p <= 10^-5^) indicating no support for the premise that bowing had this effect. From this we find that, despite the bowing effect occurring, FFPE material is appropriate for targeted methylation sequencing. Indeed, due to the nature of sequencing data (i.e. reads aligning to the genome with different starting positions) we expected an overlap of reads to the extent that no methylation event would always only be covered by bases in the first 5 – 40 bp of reads. We have no conclusive rationale as to why bowing occurs, and are very interested in the observations of other researchers on the phenomenon, and how they define and explain the effect.

### On-target and off-target sequence reads

Sequence metrics were recorded to assess data quality and are given in Supplementary Table 2. A mean of 63.97m (SD = 24.1m) total reads were sequenced per sample, with a mean loss due to filtering based on non-alignment and duplication of 25% (SD = 20%). An important determinant of good quality data was the ‘on-target’ rate, which specifies the proportion of reads sequenced that align to the genomic regions specified as the ‘target’ for sequencing, here a total of 84.5MB. The alternative to on-target is termed ‘off-target’, i.e. not aligning to the target region. Our sequence data resulted in a mean of 58% (SD = 16%) on-target reads. Therefore, to have the total targeted region covered by 1 read at every base (1x on-target coverage) we required an average of 2.5m (SD = 1.7m) total reads per sample. We had an expectation of 20% ‘off-target’ rate, but found rates in samples S1 and S3 of 53% (SDs=1, 7%) which caused a reduction in reads on-target. Sample S2 achieved the calculated 1x on-target coverage level of 0.8m total reads with almost exactly the expected off-target rate (21%, SD=7%). Interestingly, despite our low sample size, we found good concordance of off-target reads between FFPE and FF in each sample as evidenced by low standard deviations. We therefore investigated if off-target regions were the same within samples between preservation type, and also if any regions were shared between preservation types across samples.

We postulated that lower DNA quality, and possibly increased fragmentation might have caused increased off-target rates, but we did not find these phenomena to be associated. This was evident in S1_FFPE and S2_FFPE, both of which failed the β-tubulin PCR assay used to assess overall DNA quality. Full off-target results are given in Supplementary Tables 3, 4. Total off-target regions had a mean size of 737.2MB (SD = 325.4MB), with a mean intersection within samples of 263.8MB (SD = 174.3MB), equating to a range of 19-40%. Regions unique to each sample were tested between preservation types (e.g. S1_FFPE vs. S2_FFPE). However, only 5-15% of these regions were shared with either of the other samples, and no significant effect of preservation type on regions was found (Fishers Exact test, p = 0.72). We accounted for the sequencing depth by comparing average total read coverage of each sample's total off-target regions. We concluded that off-target regions were therefore not associated with preservation type, again confirming that FFPE material is not inherently biased by this effect.

Off-target reads are expected in exome data [34], another targeted sequencing approach, and are known to be spread across the genome [35], as evident in our data. All off-target reads were removed prior to further analysis, as is typical in exome analysis. The high off-target rates in samples S1 and S3 indicate that increased sequencing may be required to mitigate the impact of reduced on-target rates that result.

### C to T mutation and deamination effects

Increased deamination has previously been found associated with FFPE material [10, 12]. We tested the extent of deamination by looking for C>T mutations on the sense strand, then identifying whether the anti-sense strand was A or G, and therefore whether the mutation was a true single nucleotide variant (SNV), or whether it resulted from bisulfite treatment (see Supplementary Table 5). We found no significant increase for deamination in samples S1 and S2 (p > 0.84, 0.61 respectively). In S3, we found a significant increase in S3_FF (p < 0.006), the opposite of what would be expected if FFPE caused deamination. Our results therefore show no evidence of increased deamination in FFPE samples when compared to their FF counterparts. The significant result for S3_FF is due to an increased level of A, and therefore of called SNV, in that sample and occurs at 28% higher rate than in S3_FFPE. Interestingly, samples S1 and S2 have a G/A ratio of ca. 14%, whereas S3_FF is 24% and S3_FFPE is 20%, indicating increased rate of mutation in the sample. This may result in a broader spread of called SNV, given that more seem to exist in S3 overall.

### Pair overlap, concordance, sensitivity and validation

Having dealt with several technical issues, and not finding reason to discount FFPE material from further analysis, we profiled each individual sample using thresholds of hypo-, hyper- and intermediate methylation, defined as being <20%, >80%, and intermediate to those two values. Following this we determined concordance by intersecting profiles within samples. We used the cell line concordant profile as a ‘gold-standard’, against which we compared the tumor samples to determine sensitivity, and selected a variety of methylation events for validation using a different platform to ensure accuracy of our data.

### Initial profile characterization

**Figure 3:**
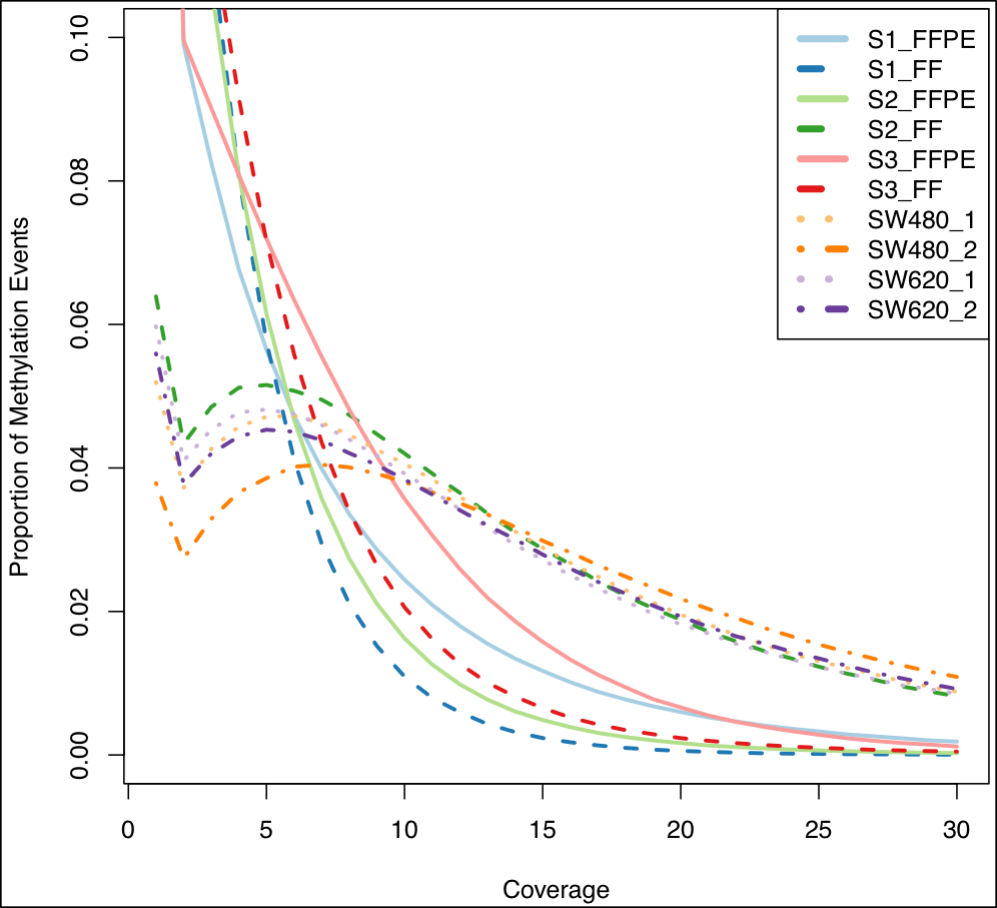
Coverage and proportion of methylation events FFPE (full lines) and fresh frozen (dashed lines) samples showed a similar issue with a high proportion of methylation events being called at relatively low coverage (~2% are above 10x); S2 fresh frozen sample (green dashed line) was the only tissue sample to resemble the cell line duplicate curves (purple, orange dotted and dot-dash lines) which we took as the gold-standard due to high quality input DNA.

Figure 3 shows the proportion of methylation events at different coverage levels per sample. Only S2_FF had a similar distribution when compared to the cell lines and so this sample was considered to be of high quality. We therefore compared the profiles of S2_FFPE and S2_FF to determine the impact on intersection. We found a higher proportion and the largest divergence in hypo-methylated events (63% in FFPE vs. 48% in FF) with similar levels of hyper-methylation (22% in FFPE and 15% in FF). Our use of inflexible thresholds, required for this broad overview of similarity, may have caused the discrepancy, as intermediate events (15% in FFPE and 25% in FF) if redistributed could ‘balance’ the observed levels of hypo-methylation. Divergence was in the range of 1-8% in the other samples and we therefore concluded that use of the S2 sample was appropriate.

### Cell lines

Pair intersection data is shown in Supplementary Table 6. More than 96% of methylation events were found in each of SW480 and SW620 between replicates (N1, N2). This proportion is mirrored when replicates were filtered to include only events with 10x or greater coverage, the results of which are stated here. For concordance, we found 86%, 92%, and 70% respectively between replicates for hypo-, hyper- and intermediate categories, indicating again that our inflexible thresholds, and especially the intermediate category, might reasonably be seen to be reducing concordance. Hypo-, hyper- and intermediate categories made up 36, 22 and 42% of the total intersecting events. These results were of interest because we expected a very high level of reproducibility in cell lines, but still found a relatively high level of discordance, particularly given our 10x coverage filter.

### FFPE-FF samples

Pair intersection data is available in Supplementary Tables 7, 8. We found 84, 84 and 95% of methylation events extant in the intersection of FFPE and FF in S1, S2 and S3. Of these, just under half were concordant based on our thresholds for hypo- hyper- and intermediate methylation (49, 43 and 43% per sample respectively). Interestingly, the majority of concordant events were hypo-methylated (37, 28 and 29%), with approximately 1/8^th^ of events concordant and hyper-methylated (12, 15 and 14%).

### Discordant events

**Figure 4:**
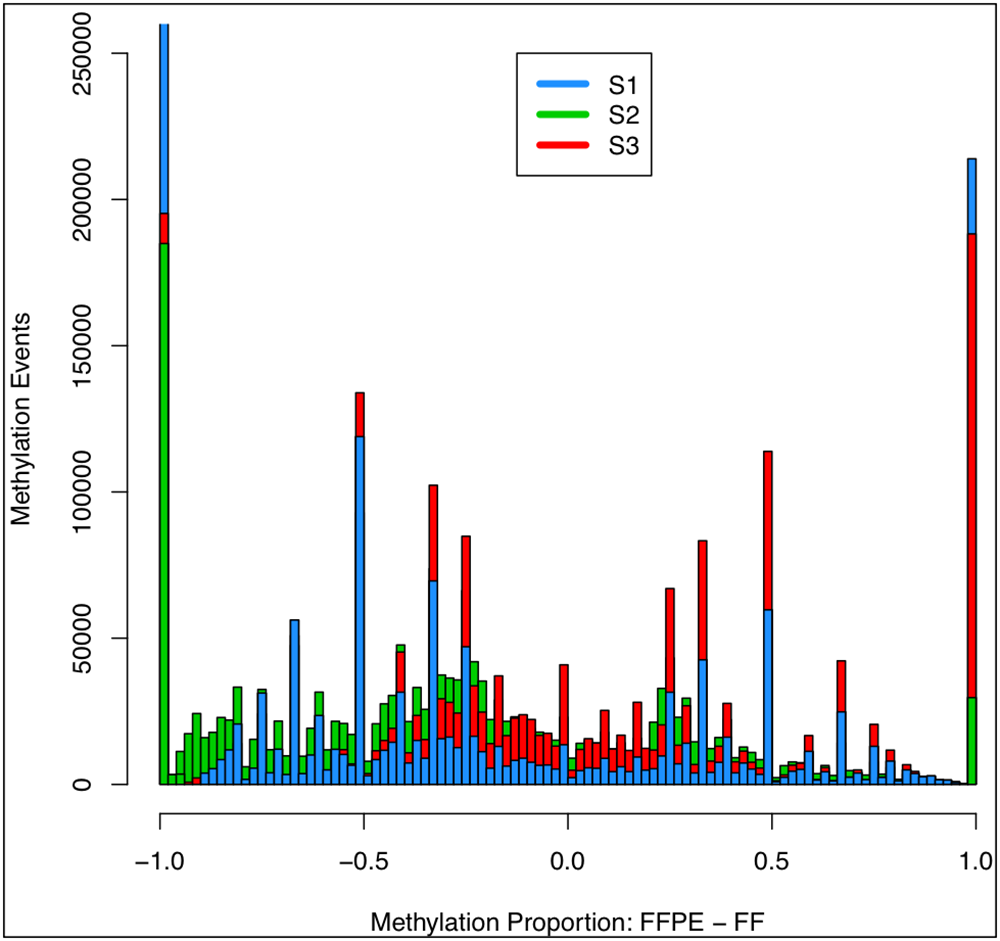
Discordant methylation events between FFPE and fresh frozen samples Methylation events found not to be concordant between FFPE and fresh frozen tissue samples from the same patient had methylated proportions subtracted (FFPE-fresh frozen, x-axis, 0.02 bins); negative values indicated higher level of methylation in fresh frozen samples, largely apparent in S2 sample, previously found to have a high quality fresh frozen sample (see Figure 3); the majority of discordant events occurred at -1, 1, highlighting that complete divergence (one sample fully methylated, the other not at all) is the most frequent, but in both FFPE and fresh frozen this effect is likely to occur from low coverage.

Figure 4 shows the distribution of discordant events between preservation types within samples. Immediately evident was that the majority of discordant events occurred at the tails, i.e. when methylation is total in one preservation type and absent in the other. This is likely due to low coverage, where an aberrant result (either real or technical) would be seen as either 1 or -1 (methylated in FF and not FFPE, or vice versa, respectively). Interestingly, methylation in FFPE had fewer events at which there was a higher level of methylation than compared to FF. This effect is most pronounced in S2, which we viewed as the most divergent pairing given the previously reported high quality of S2_FF. Higher coverage is likely to be the cause of the distribution effect, and increased sequence depth would control this.

### Sensitivity

**Figure 5:**
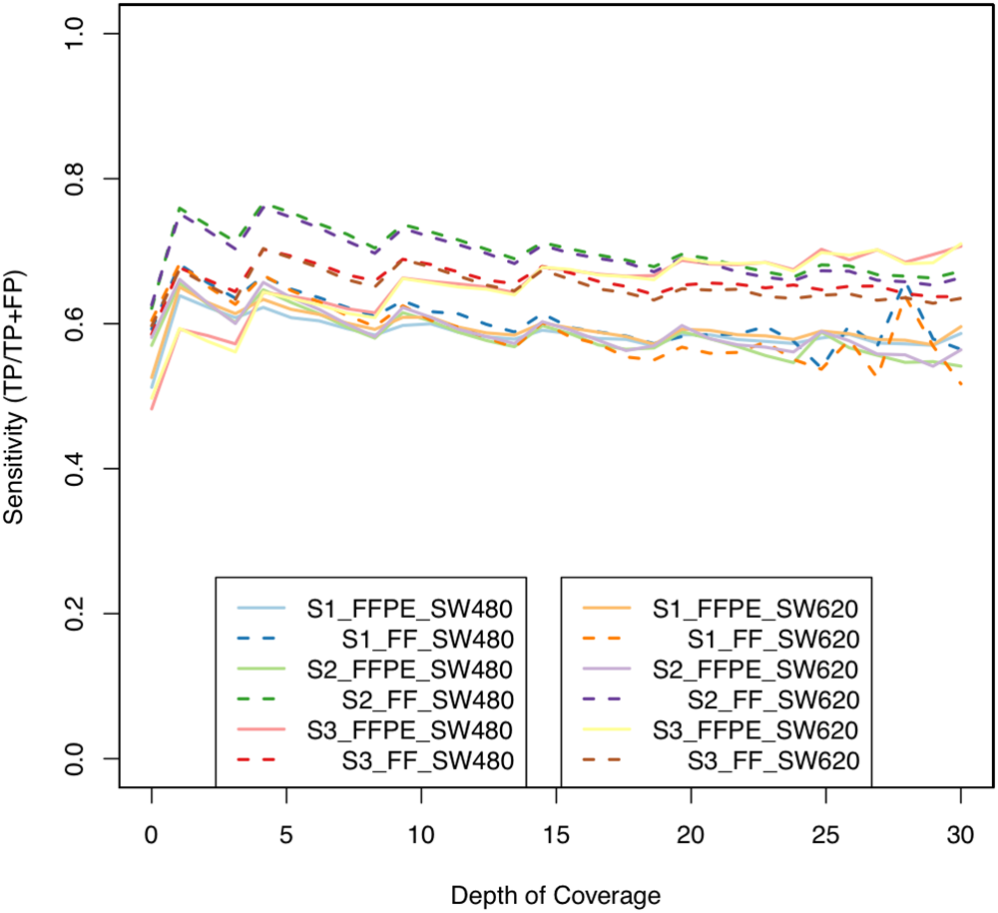
Sensitivity versus coverage Sensitivity was determined using the cell line data (SW480, SW620) by finding events in which samples agreed with cell lines; fresh frozen samples (dashed lines) appeared marginally better (S2 in particular again) at low coverage, but with increased coverage, FFPE samples (full lines) became relatively similar, if not better in the other FFPE samples (e.g. S3, red/yellow full lines); increasing coverage did not dramatically increase sensitivity, and so we did not expect conservative filtering to result in more accurate data.

We determined sensitivity based on a set of ‘true positives’ (TPs). These were defined as the concordant intersection of cell lines at 10x coverage. One profile was generated for SW480 and one for SW620. These profiles included approximately 50% of events in the intersection of each of the sample profiles, retaining approximately 3 million methylation events. Figure 5 shows sensitivity rate at coverage from 1x to 30x. We found that moving from 1x to 2x coverage increased the likelihood of TP calls by at least 7% in all samples, and that by and large FFPE and FF samples maintained a similar TP rate thereafter. The exception was S2_FF, and we believe this again to be due to the distribution of methylation events described earlier which are indicative of a high quality sample. The FFPE samples suffered from a reduced sensitivity versus FF, plateauing around 60% for S1_FFPE and S2_FFPE. Interestingly S3_FFPE, which at low coverage had low sensitivity, gradually increased to the highest rate and was very close to S3_FF from 10x on. S1_FFPE and S1_FF both had low sensitivity, indicating poor sample quality overall. An obvious caveat worth repeating is that cell lines do not necessarily mirror tumor tissue and so we should not expect very high sensitivity. Their use here is in a technical capacity knowing the DNA quality to be very good.

### Validation

We were able to validate 5 regions using the Sequenom Epityper platform (Supplementary Figures 2a-2e). These regions were chosen based on being TP or FP, and also for having hyper-, hypo- and intermediate methylation levels. The maximum deviation between methylation sequence data (MSQ) and the Epityper (EPI) was 0.4 in two regions but these were both in SW620 cell line, again highlighting that these lines do not absolutely represent tumor. One region, the first on chr13 (Supplementary Figure 1c), showed consistently lower calls from MSQ by ca. 20% (0 vs. 20% methylation on EPI). This effect was evident across S1_FFPE, S1_FF and S2_FFPE, with S2_FF the only sample showing high concordance between MSQ and EPI. All other regions validated well and show that the sequence data used was accurate to the extent that these regions could determine.

## MATERIALS AND METHODS

### DNA extraction and quality assessment

The experimental design consisted of DNA extraction from two different sources of clinical material – FFPE and FF tissue – from 3 patients (retrospective clinical samples collected as a part of FP7-funded initiative *ANGIOPREDICT*), in addition to two colorectal cell lines in duplicate, SW480 and SW620 (total n=10) (Figure 1). The cell lines used for this study were obtained from the American Type Culture Collection (ATCC), and grown in the recommended conditions (L-15 media + 5% FBS+ Penicilin/Streptomycin, 37°C and 5% CO_2_). In the case of FFPE samples, DNA extraction was carried out following deparaffinization of 3 × 20μm sections using the Qiagen FFPE-DNA extraction kit. Sections were de-waxed through high-heat incubation (60°C for 1 hr) followed by serial dehydration through exposure of to varying ethanol concentrations (100% ethanol x 3, 90% ethanol x 2, 80% ethanol x 1, 70% ethanol x 1; all exposures for 1 min each). Subsequently, sections were rehydrated using water (water x 3, 1 min each). Once rehydrated, sections were scraped into a microcentrifuge tube and incubated overnight with proteinase-K and digestion buffer at 56°C. Following incubation, DNA was extracted as per the manufacturer's instructions. For DNA extraction from FF material, the tissue was initially macerated using a scalpel in order to homogenize the tissue. The tissue was then incubated with proteinase-K and digestion buffer. Following this, the DNA was extracted using the Qiagen DNAeasy blood and tissue kit as per manufacturer's instructions. Similarly, DNA was extracted from the two colorectal cell lines using the aforementioned kit. The double-stranded (dsDNA) concentration was determined using Picogreen (Invitrogen). DNA quality in terms of level of fragmentation was assessed using gene-specific PCR-based analysis. For each sample, two PCR reactions were carried out using primers specific for GAPDH (Forward: ATGGGGAAGGTGAAGGTCG, Reverse: GGGGTCATTGATGGCAACAAT - 100bp amplicon) and β-Globin (Forward: GGGTTTGAAGTCCAACTCCTAAG, Reverse: CAACTTCATCCACGTTCACCT - 300bp amplicon). Following the reaction, the PCR products were analyzed using agarose gel-electrophoresis. The presence of a band for a given sample at the desired size was determined as passing the quality threshold.

### DNA library preparation, quality control and sequence capture

An input of 500ng of dsDNA was used to generate DNA libraries using the standard Kapa LT-DNA library preparation kit (Kapa Biosystems, KK8320). The libraries were assessed for quality using high-sensitivity DNA Bioanalyzer chips (Agilent Technologies) along with quantification using Picogreen. Bisulfite conversion was conducted using the Zymo DNA methylation lightning kit (Zymo Research). 500 ng of quantified DNA library was processed using the CpGiant SeqCapEpi capture kit (Roche Nimblegen) as described previously [32]. Briefly, 500 ng of the bisulfite treated DNA library was incubated with the sequence capture probes for 72 hrs at 47°C. This was followed by incubation of probe and library mix with streptavidin beads for 45 mins at47°C with frequent mixing. This would allow the beads to selectively bind to the biotin labeled probes. Subsequent washes with buffers: Stringent wash buffer, wash buffer I, II and III at temperatures and wash times as per manufacturers instructions. The captures sample was amplified using LM-PCR and assessed for overall quality using a High-sensitivity bioanalyzer chip (Agilent Ltd.). Finally, the captured libraries were sequenced using a paired end 125 bp strategy on Illumina HiSeq 2000 v4.0. Data can be accessed from the Gene Expression Omnibus, accession number GSE84171.

### Trimming, alignment and methylation calling

We used the BBDuk trimming method from the BBMap package (version 35.14, [36]) to trim adapters and remove low quality bases (Phred < 20). A kmer value –*k*=31, the size of the largest adapter, was used and a minimum kmer value –*mink*=5 applied to the 3’ end of reads.

Alignment to the hg19 genome was conducted using bwa-meth [33]. The BAM file had readgroup information added and was reordered based on chromosomal order of the reference genome, following which duplicate reads were removed. These processes were conducted using Picard-tools AddOrReplaceReadGroups, ReorderSam and MarkDuplicates respectively (v1.95, [37]). The BAM file was sorted and indexed using SAMtools (v1.2, [38]).

BisSNP [39] was used to realign reads to known indels using the Mills and 1000 Genomes ‘gold standard’ downloaded in the GATK resources bundle from the Broad Institute's public FTP server (tinyurl.com/GATK-res). Recalibration was conducted as default except with –*maxQ*=40. BisulfiteCountCovariates was used before and after recalibration to allow visualization of the effect of recalibration. BamUtil clipOverlap tool (http://genome.sph.umich.edu/wiki/BamUtil:_clipOverlap) was used to clip overlapping sequence shared by paired-end reads, again resulting from short insert sizes in libraries.

BisSNP BisulfiteGenotyper was used to call CG events and SNPs. We applied a filter of minimum quality –*mmq*=30 and also restricted calls in the first and last 5bp of reads –*trim3*, *5*=5 following inspection of bias-plots which were generated following alignment and also after clipping overlaps. We also used a range of 5 – 90 bp trimming from 5’ as detailed (see Results) to determine the effect that this might have on the bowing effect evident in FFPE samples. Finally, methylation profiles consisting of single base resolution calls were filtered using BisSNP VCFPostProcess and converted to BED format using in-house Perl scripts. Intersection of individual methylation event sets was then conducted using the BEDtools [40] package and in-house Perl scripts.

Thresholds for methylation events were used for concordance, with events showing total C of less than 20% being called as hypo-methylated, and similarly hyper-methylated were determine as having greater than 80% C calls at the base. Finally, an intermediate level of between 20-80% C base at a position was used.

### C to T mutation rates

To determine the level of deamination, thought to be an induced feature of FFPE tumor material, C>T transitions were investigated using an in-house Perl script (https://raw.githubusercontent.com/brucemoran/perl-tools/master/BisSNP_snp_C2T.pl) using all C>T SNPs called by BisSNP as input. It was determined if the opposite strand at the SNP position was a real mutation (A) or an unmethylated cytosine (G) caused by bisulfite treatment. Increased adenosine in FFPE versus FF would suggest increased deamination. We removed the first and last 10bp of reads in BAM files due to lower base quality, as per BisulfiteGenotyper. Only primary alignments were used. We next used a Fisher's exact test on a 2×2 table for A or G counts in the FFPE and FF samples to determine if preservation method effected C>T rate.

### Intersection of cell lines and FFPE-FF pairs, consensus and concordance

To determine if and how preservation method impacted methylation, we used BEDTools intersect method [40] to intersect profiles for each of the three FFPE-FF pairs and the two replicates of each of the SW480 and SW620 cell lines, noting the total methylation calls made and the proportional overlap found. The output from this step was what we termed the ‘consensus’ profile, i.e. the consensus methylation at the sample level. We also used what we term a ‘concordance’ approach. Thresholds of methylation in ranges 0-20%, 21-79%, and 80-100% were defined as hypo-, intermediate or hyper-methylated respectively. We then investigated concordance of these categories between FFPE and FF, and between cell line consensus and patient samples. Whilst this approach might not incorporate the subtlety of intermediate levels of methylation, this study was specifically focused on how well FFPE mirrored the methylation events in FF material, and so categorization was intrinsically useful to this end. For consensus profiles in cell lines, a fourth category, ‘off’ was used to denote where the replicates were not concordant. These positions were removed for the sensitivity analysis so as not to bias results from FFPE and FF material.

Due to the exploratory nature of the study, we did not impose a coverage filter on patient samples. A 10x cutoff was imposed for cell lines based on preliminary results indicating that similar proportions of each category of methylation level for 1x and 10x coverage were apparent. Using a higher coverage threshold allowed extra confidence when comparing cell lines with patient samples, a key reason for using the cell lines in the first instance. To determine if a pattern of discordant events existed, i.e. methylation was called in one category in FFPE and another in FF, we plotted the distribution of the discordant events in the three tissue sample pairs taking a scale from -1 to 1, where -1 indicated total methylation in FFPE and no methylation in FF, and 1 indicated total methylation in FF and no methylation in FFPE.

### Sensitivity analysis

We created a ‘true positive’ profile in cell lines, consisting of the intersection of SW480 and SW620 consensus profiles. We used this true positive cell line profile to determine false positive (FP; when cell line event call differed from patient samples) and true positive (TP; when cell line event call was concordant with patient samples) in FFPE and FF. We determined the distribution of sensitivity, the rate of true-positives defined as TP/(TP+FP), at the range of coverage evident in FFPE-FF consensus profiles. The results generated using these approaches illustrate the overall concordance in methylation events between the cell lines and the patient samples, therefore indicating performance of FFPE compared to FF. The data generated from this analysis was subsequently used to select targets for validation using an independent platform.

### Validation targets

DNA was unavailable for validation from S3, and so the validation was conducted on S1 and S2 pairs only. Validation targets were selected to test for a variety of different possible situations in the context of concordance of matched patient pairs and the cell lines to be validated using the EpiTYPER validation approach as described by [40]. DNA corresponding to each of the samples was bisulfite treated, followed by PCR amplification as per manufacturers instructions (Agena Biosciences). This was followed by SAP digestion, T-cleavage transcription/ RNAase A incubation, followed by dispensing the samples in the SpectroCHIP® array to be assessed using the Sequenom instrument. Each region to be validated was selected based on having at least 10x coverage in all FFPE and FF samples, as well as either being TP or FP in consensus profiles of both cell lines. We subdivided methylation events in those categories into being found unique to either S1, in both S1 and S2 but not S3, or being found in all three matched sample pairs. Initially, we determined 9 regions for validation, but primer design failed for 4 and so the final validation set contained 5 regions.

## CONCLUSION

Global methylation profiling of DNA isolated from FFPE tissue is presumed to present challenges potentially owing to the overall quality as well as associated artifacts. Our study has generated single-base resolution DNA methylation profiles for specific regions of interest. This equated to approximately 5.5 million CpG sites across the genome. We compared profiles between matched FFPE and FF samples to examine the impact of FFPE-linked artifacts on methylation calls. We have shown that a targeted NGS methodology is applicable to tissues originating from different preservation types, and no serious inadequacy has been shown in either of FFPE or FF. The main source of concern for future work would be overall sample quality, as seen in the lower sensitivity of both preservation types in sample S1. The targeted approach allowed extrapolation of C>T transition artifacts previously associated with FFPE [23, 25]. We have found no increase in such artifacts.

The finding that the bowing pattern did not impact on the FFPE samples is relevant in the context of the study. It again highlights that, despite an effect specific to FFPE, no associated reduction in correlation points to sample-specific conditions as the more relevant confounding factor during DNA methylation analysis. To our knowledge, this issue has not been fully investigated in targeted methylation sequencing previously. The approaches used allowed identification of true and false positives which were validated on an independent platform, thus substantiating the efficacy of the NGS methodology. However, our results show inherent noise based on our analytical approaches. Primarily, defining thresholds of methylation, i.e. for hyper- and hypo-methylation, resulted in increased discordance of results. A better approach for functional analysis would be to define a ‘distance’ (i.e. a change of 50% or more) from which one could infer or even validate potential activity. Coupled with a high off-target rate, we feel it is important to reinforce that increased levels of sequence data, or indeed a reduced target region size specific to the disease type being investigated, would be of benefit to similar studies based on our findings. This study highlights some key issues associated with FFPE derived material and we hope they will be taken into consideration by other researchers designing targeted methylation sequencing experiments.

## SUPPLEMENTARY MATERIALS FIGURES AND TABLES


















