# Supplementary material for: Assessment of concordance between fresh-frozen and formalin-fixed paraffin embedded tumor DNA methylation using a targeted sequencing approach
Source: Oncotarget. 2017 May 30;8(29):48126–37. doi: 10.18632/oncotarget.18296 (PMC5564631; doi:10.18632/oncotarget.18296)
Supplement: Supplementary file 1 [file oncotarget-08-48126-s001.pdf]

Assessment of concordance between fresh-frozen and formalin-fixed paraffin embedded tumor DNA methylation using a targeted sequencing approach

SUPPLEMENTARY MATERIALS

SUPPLEMENTARY FIGURES AND TABLES

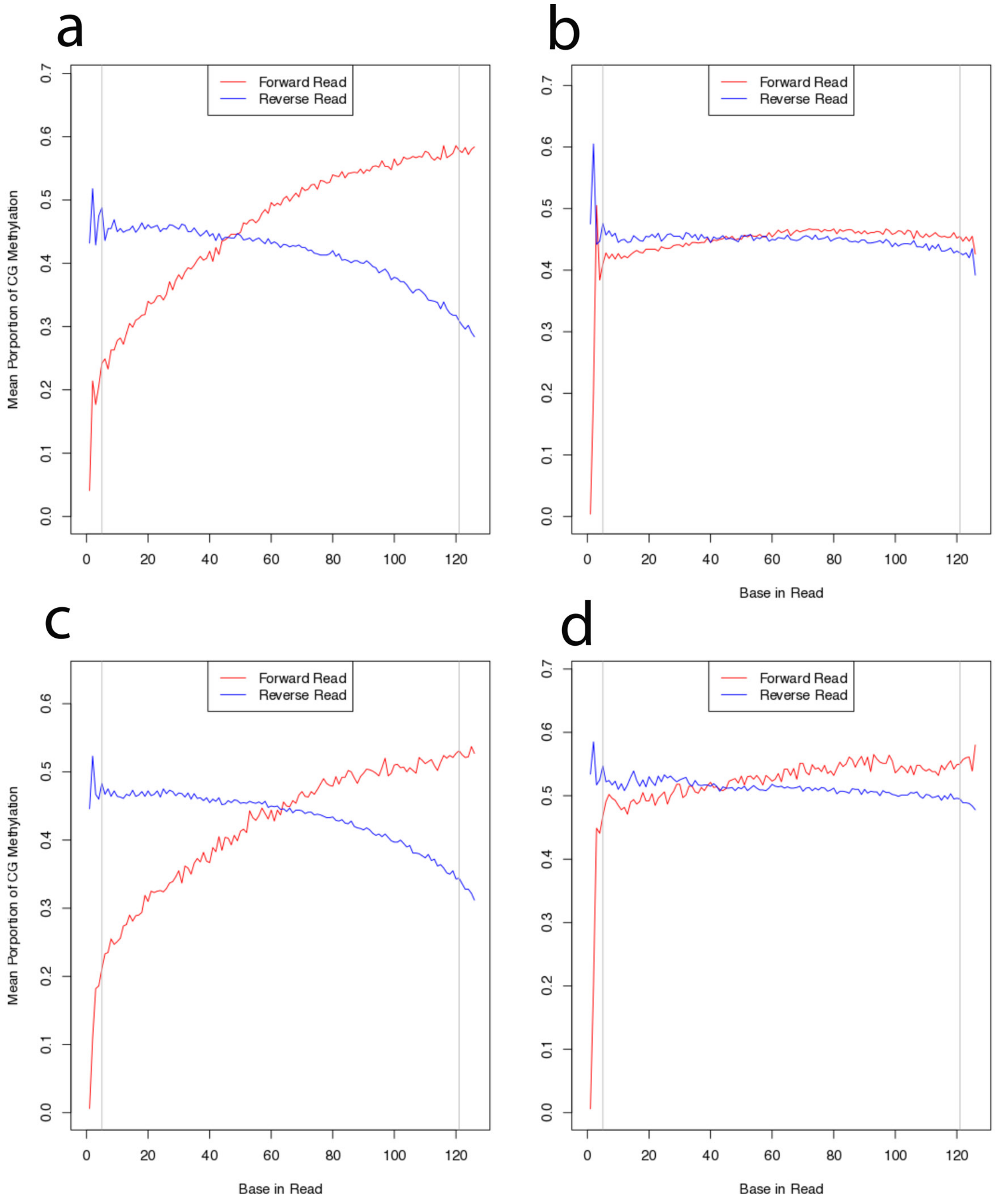

Supplementary Figure 1: Methylation Bias Plots of Bowing Effect in Samples S2, S3. Mean CG proportion per base of sequence reads in: **(a)**, sample S1_FFPE; **(b)**, sample S1_FF; **(c)**, sample S3_FFPE; **(d)**, sample S3_FF.

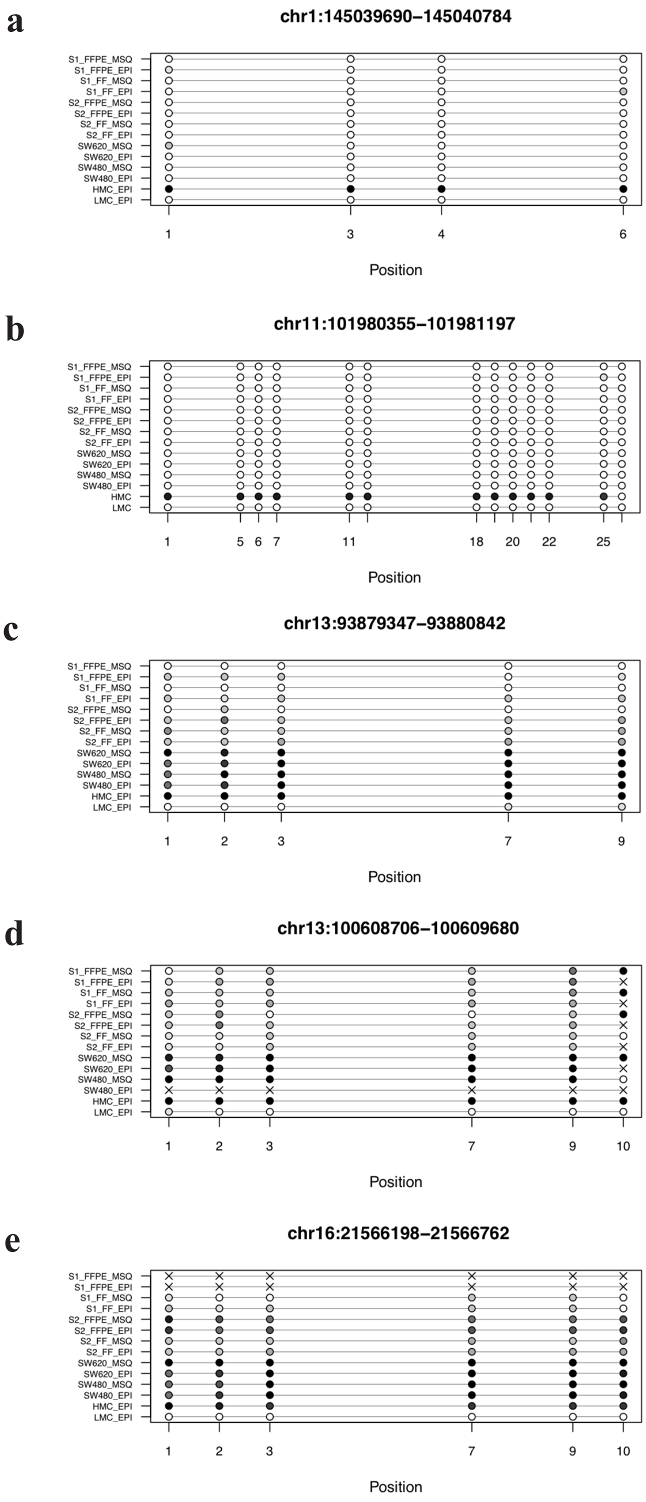

Supplementary Figure 2: Sequenom Epityper (EPI) Validation of Methylation Sequencing (MSQ). Plots show dots, shaded by increased methylation; FFPE and FF samples for S1, S2, and SW480 and SW620, high methylation control (HMC) and low methylation control (LMC) are show top to bottom; **(a)** concordant at all positions, HMC at 100% methylated; **(b)** concordant at all positions, HMC at 100% methylated; **(c)** high methylation levels in cell-lines, low levels in samples; EPI validation S1, S2 shows increased levels but not to the range of cell lines; **(d)** high levels in cell lines, intermediate levels in samples S1, S2 concordant for EPI, MSQ; final position failed on EPI; **(e)** high methylation levels in cell-lines, divergence in sample S2 between FFPE (elevated) and FF (intermediate); S1 FFPE failed, but S1 FF showed same trend as S2 FF (intermediate).

Supplementary Table S1: Samples S1-S3 for both tissue preservation types were trimmed at 5, 30 and 60 bp and had methylation events called. The FFPE and FF calls were intersected (overlap) to show level of concordance; three hard filters for 1x, 2x and 4x reads per methylation event were investigated to determine if this was affected by bowing

See Supplementary File 1

Supplementary Table S2: Sequencing metrics per sample and tissue preservation type. Total reads, aligning, following duplicate removal, and proportions on-target are shown, with specific coverage on-target also presented

See Supplementary File 2

Supplementary Table S3: Off-target metrics per sample indicate large regions are represented by off-target reads

See Supplementary File 3

Supplementary Table S4: Per-chromosome off-target read totals and proportions give no evidence for specific chromosomal effects

See Supplementary File 4

Supplementary Table S5: Levels of 'A' and 'G' calls on the opposite-strand to 'C' indicate no increased deamination in FFPE samples

See Supplementary File 5

Supplementary Table S6: Cell line methylation call metrics and proportional intersection at thresholded methylation events

See Supplementary File 6

Supplementary Table S7: FFPE and FF sample methylation call metrics

See Supplementary File 7

Supplementary Table S8: Patient sample-level methylation call metrics

See Supplementary File 8
